# Supplementary material for: When optimization for governing human-environment tipping elements is neither sustainable nor safe
Source: Nat Commun. 2018 Jun 15;9:2354. doi: 10.1038/s41467-018-04738-z (PMC6003916; doi:10.1038/s41467-018-04738-z)
Supplement: Supplementary file 1 — Supplementary Information [file 41467_2018_4738_MOESM1_ESM.pdf]

Supplementary Information

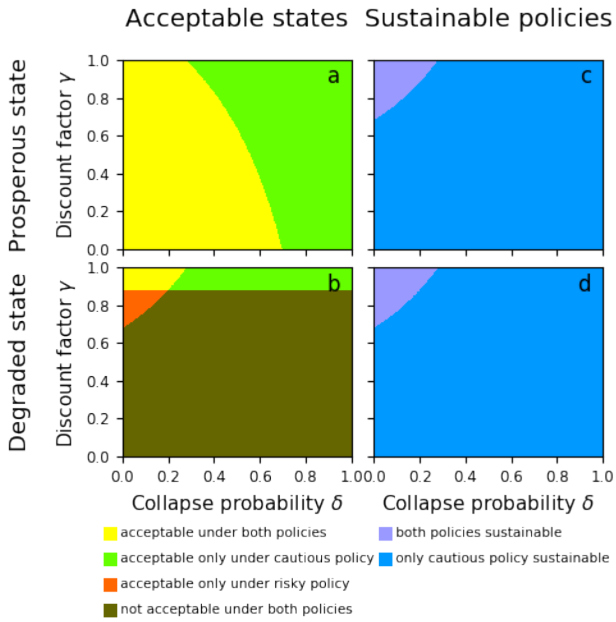

Supplementary Figure 1: **Sustainable policies are based on acceptable states** as illustrated here in the parameter space (shown as collapse probability δ vs. discount factor γ with the prosperous state (a,c) and the degraded state (b,d)). Parameters are $\rho = 0.2$, $r_l/r_h = 0.5, r_{min}/r_h = 0.3$. In (a,b) color indicates whether the respective state is acceptable under different policies. In (c,d) color indicates the resulting sustainable policies.

	Climate	Fishery	Farming
Assumed collapse timescale [years]	~ 40	~ 20	~ 100
Corresponding collapse probability	~ 0.025	~ 0.045	~ 0.01
Assumed recovery timescale [years]	$\to\sim \infty$	~ 50	~ 300
Corresponding recovery probability	$\to\sim 0$	~ 0.02	~ 0.003

Supplementary Table 1: Typical transition timescales and corresponding probabilities.
